# Optical Polarization Sensitive Ultra-Fast Switching and Photo-Electrical Device

**DOI:** 10.3390/nano9121743

**Published:** 2019-12-07

**Authors:** Jérémy Belhassen, Zeev Zalevsky, Avi Karsenty

**Affiliations:** 1 Grenoble Institute of Technology (Grenoble INP), Photonics and Semiconductors Section, Grenoble-Alpes University (UGA), 38400 Saint-Martin-d’Hères, France; jerem.belhassen@gmail.com; 2 Advanced Laboratory of Electro-Optics (ALEO), Department of Applied Physics/Electro-Optics Engineering, Faculty of Engineering, Lev Academic Center, Jerusalem 9116001, Israel; 3 Faculty of Engineering, Bar-Ilan University, Ramat Gan 52900, Israel; zalevsz@biu.ac.il; 4 The Nanotechnology Center, Bar-Ilan University, Ramat Gan 52900, Israel; 5 The Nanotechnology Center for Education and Research, Lev Academic Center, Jerusalem 911600, Israel

**Keywords:** optical switching, Encryption, decryption, V-groove, nanoscale optical modulator, electro-optical coupling, polarization, simulations, evanescent waves, wavelength aperture

## Abstract

Ultra-fast electrical switches activated with an optical-polarized light trigger, also called photo-polarized activated electrical switches, are presented. A set of new transistor circuits is switched by light from above, illuminating deep V-grooves, whose angle is sensitive to the polarization of the incident. Thus, this application may serve for encryption/decryption devices since the strongest electrical responsivity is only obtained for very specific spatial polarization directions of the illumination beam. When this V-groove is sufficiently narrow, the device mainly responds to one polarization and not to the other. In such a way, electrons are generated only for one specific polarization. While the nature of the data remains electronic, the modulation control is optic, creating a photo-induced current depending on the polarization direction. This coupled device acts as a polarization modulator as well as an intensity modulator. The article focuses on the integration of several devices in different configurations of circuitry: dual, triple, and multi-element. Case studies of several adjacent devices are presented with varying critical variables, such as the V-groove aperture dimensions. Analytical models and complementary numerical analyses are presented for the future smooth integration into Complementary Metal-Oxide-Semiconductor (CMOS) technology.

## 1. Introduction

### 1.1. Ultra-Fast Electro-Optic Polarized Switch

The need for ultra-fast optical switching methods is continuously growing [1,2]. Several techniques were developed in the last decades, some of which were based on the electro-optic effect [3]. Most of these techniques use special materials, such as Potassium Lithium Tantalum Niobate (KLTN) crystals [4]. Others are based on molecules [5] or Gallium phosphide (GaP) [6]. One can now imagine how useful the switching effect for micro-electronic needs could be when using a silicon-based device.

A new approach is now presented using a selective polarization phenomenon in coupled devices, and sharing an electro-optic trigger with micro-electronic data transfer. We developed Polarizing Transistors (PT), also called Silicon-On-Insulator Photo-Polarized Activated Modulators (SOIP^2^AM) that share a V-groove component. When this V-groove is sufficiently narrow, the device mainly responds to one polarization direction, and not to the other. In such a way, electrons can be generated only for one specific polarization. While the nature of the data remains electronic, the modulation control is optic, creating a photo-induced current pending the polarization direction. The uniqueness of this coupled device is that it acts as a polarization modulator as well as an intensity modulator. This innovative device is the product of a continuous process keeping the same architecture of a V-groove geometry, and changing the triggering modulation starting from illumination activation [7,8,9,10,11], continuing to thermal activation [12,13], recently progressing with polarization activation [14], and now embedded in a series of adjacent devices with selective polarization. This is the reason why, in parallel to its evolution, this special modulator changed its name from SOIPAM (Silicon-On-Insulator Photo-Activated Modulator), to SOITAM (Silicon-On-Insulator Thermo-Activated Modulator), SOIP2AM (Silicon-On-Insulator Photo-Polarized-Activated Modulator), and N-SOIP2AM for multi-elements (N) integration. The dimensions have evolved as well—whereas preliminary versions were shaped in micrometric proportions, the most recent ones are already shaped in nanoscale dimensions. Table 1 summarizes the main critical parameters.

### 1.2. The Original Photo-Electrical Device Concept

**Figure 1:**
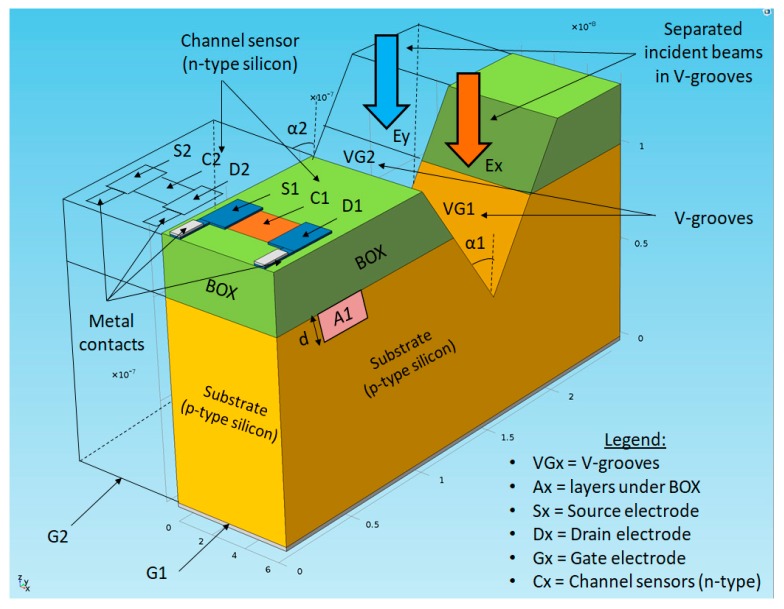
Schematic representation of the dual-SOIP^2^AM devices with separated incident polarized beams.

As presented in the past, the original SOIPAM device is composed of a 30 nm deep n-doped channel (donors concentration N_D_ ~ 1.10^17^ cm^−3^) located between a Source and a Drain (Source-Drain distance is L _Channel_), like a Metal-Oxide-Semiconductor Field-Effect-Transistor (MOSFET). Under this channel, a 150 nm thick buried oxide (BOX) layer separates the channel from the p-doped substrate (N_A_ ~ 1.10^15^ cm^−3^). A negative voltage V_GS_ is applied under the substrate, which will draw the holes (majority carriers) backward from the substrate, leaving a negative depletion layer under the oxide. By electrostatic effects, a positive depletion layer is created in the n-channel, just above the oxide. In dark conditions, the channel is designed to still be open (ON), despite this depletion. Under illumination, the photo-generation phenomenon creates electron-hole pairs in the substrate, which will be separated by the electric field due to the voltage V_GS_. The photo-generated electrons will then contribute to creating an inversion layer under the oxide, causing an electrostatic effect, an expansion of the depleted layer in the channel until the closing of the channel. SOIPAM’s operation is, therefore, close to an inverted MOSFET (because of its bottom negative V_GS_ voltage) with optical control of the channel closure rather than an electrical control. The challenge is to succeed, by illuminating the substrate, to create enough electron-hole pairs in the substrate, which is not easily reachable by illumination. Therefore, the proposed solution was to create a V-groove (Figure 1 and Table 1, allowing a direct illumination of the substrate. The V-groove was placed beside the, and the carriers diffused throughout the sample as our model showed. This time the V-groove will be used for the selective polarization control of the channel’s closure. In this paper, the performance of two and three adjacent elements will be investigated.

### 1.3. Modeling and Fabrication Parameters

In the fabricated device, as well as in the modeling parameters, we used a very thin n-type Silicon-On-Insulator (SOI) layer with a p-type substrate. The starting material was an SOI wafer with a silicon layer thickness of about 30 nm and n-type doping of 10^17^ cm^−3^. The thickness of the buried oxide insulator is about 150 nm, and the p-type silicon substrate has a doping concentration of 10^15^ cm^−3^. The diffusion length is defined as:

(1) $$L\;=\;\sqrt{\frac{kT\mu \tau }{e}},$$

where *k* is Boltzmann constant, *t* is the temperature, μ is the mobility, *e* is the charge of electron, and τ is the specific lifetime. In Si, it is simple to achieve L close to 1 mm. The measurements done in the device are done by measuring the drain current, and its value during the illumination can increase by more than an order of magnitude. In the proposed device, the closed state occurs when the modulated gate voltage V_GS_ is negative. By synchronously illuminating the p-type area, free photo-generated electron concentrations can be significantly and instantly increased until the inversion state is reached. As a result, the voltage drop on the p-type silicon is reduced; consequently, the voltage drop on the n-type layer is increased. As a result, the depletion layer in the n-type channel is widely extended and eventually closes the channel. Under these conditions, the “dynamic” inversion layer is sustained by applying external illumination. That inversion layer is sustained until a positive voltage is applied to the gate contact. Once the illumination is decreased, thermal-generation takes place and eventually creates an inversion layer that could also close the channel. However, if the modulation rate of the V_GS_ is high enough, the thermal-generation would not be efficient because of its relatively long-time response constant, and the device remains in deep depletion.

### 1.4. Configurations, Key Parameters, and Corresponding Applications

#### 1.4.1. Dual-Element (II) Configuration—Control Using Data Superposition

**Figure 2:**
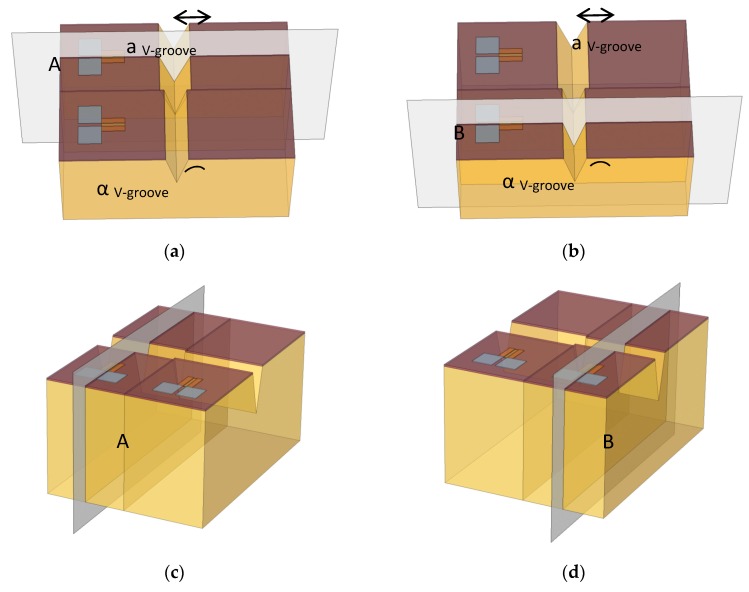
COMSOL mesh representations of the slice plans in the II-SOIP^2^AM device. (**a**) Top view with a cross-section of device A; (**b**) Top view with a cross-section of device B; (**c**) Side view with a cross-section of device A; (**d**) Side view with a cross-section of device B.

A possible application of the device would be to enable two types of data with the same light source. Indeed, assuming a single light source brings one type of data in one polarization direction, and another type of data in the perpendicular direction, two SOIP^2^AM devices. Therefore, two different V-grooves can discriminate between the relevant information. Moreover, one could independently control two elements, using only one light source. Adjacent devices can be placed in several configurations, parallel (as presented in Figure 1 and Figure 2), or perpendicular. Assuming that the first device shares an X-direction opening V-groove, and the second one shares a Y-direction opening V-groove, the OFF mode—e.g., the closure of the channel—of the first device (respective of the second one), can be obtained by applying a relevant illumination, polarized in the X-direction or Y-direction. To close both devices, one has to apply an illumination polarized both in the X and Y directions, meaning a circularly polarized illumination.

#### 1.4.2. Triple-Element (III) Configuration—Complex Binary Structure

**Figure 3:**
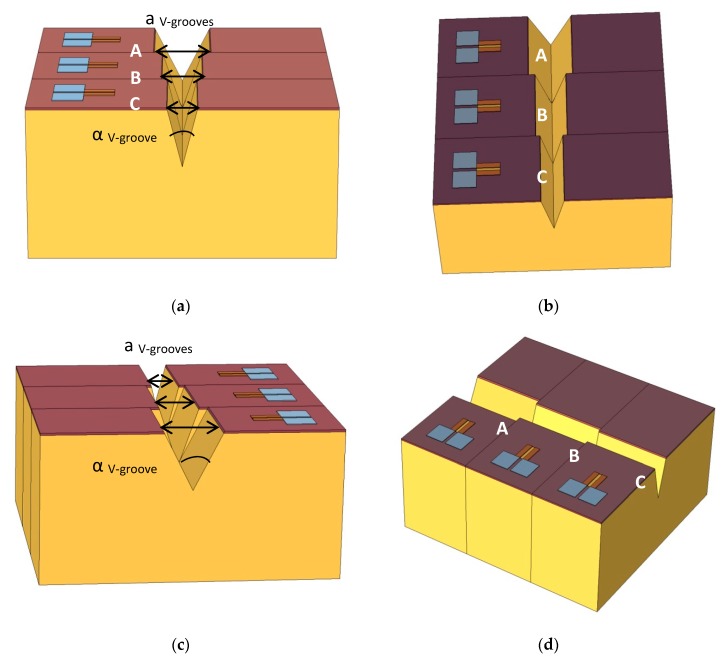
COMSOL structure representations of the III-SOIP^2^AM device. (**a**) Front view; (**b**) Top view; (**c**) Cross view; (**d**) Side view.

**Figure 4:**
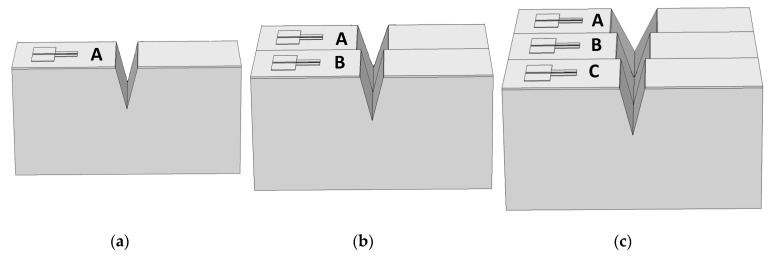
COMSOL numerical simulations of several devices sharing different V-groove apertures. (**a**) Single SOIP^2^AM (α_1_ = 28.48°); (**b**) Dual SOIP^2^AM (α_1_ = 28.48° and α_2_ = 53.83°); (**c**) Triple SOIP^2^AM (α_1_ = 28.48°, α_2_ = 53.83°, and α_3_ = 74.57°).

As previously published [14], the critical feature of SOIPAM is the opening angle of the V-groove. In the first generations of fabricated micrometric SOIPAM, the V-groove was etched by an anisotropic wet KOH technique [15,16]. Towards the fabrication of more advanced generations, the simulation of several angles and the measurement of the concentration of electrons in a defined area below the BOX has been proposed, as it has been done to date. In this kind of simulation, the COMSOL software does not accept angles as inputs, but only the size of the V-groove opening. This is the reason why fixed aperture sizes are then translated to angle values, and not vice-versa. In this study, the simulation results for the V-groove apertures of a _V-groove_ = 2 μm, 4 μm, and 6 μm, resulted in angles of α _V-groove_ 28.48°, 53.83°, and 74.57°, respectively (Figure 3 and Figure 4). The values were chosen to fit the diameter of existing commercial laser beams, in addition to fitting a constant depth of the V-groove, since the angle is determined by two dimensions. Therefore, simulating and fabricating adjacent V-grooves, sharing different opening angles, so one can fix in advance the one that could be switched to the OFF mode, seems desirable.

#### 1.4.3. Multi-Elements (N) Configuration—Polarizing Transistors Circuitry

Moving forward from a simple case study of two adjacent SOIP^2^AM devices, one can now imagine a more complex configuration of circuitry, using a series of adjacent elements, all sharing parallel V-groove parts but with different aperture values. Such a case study can be interesting for several important applications; a first application would be to enable the sensitivity to the wavelength, meaning the V-groove can be designed to filter specific wavelengths. For example, in a configuration of three elements, a blue wavelength closes only one device (OFF mode) when compared to the red wavelength, which closes all the three devices. A second application consists of being reactive to the polarization, e.g., the same case study as presented above, but with sensitivity to the polarization direction. For example, an incident illumination beam sharing a perpendicular polarization direction will enable the OFF mode of one device, when the parallel one enables the OFF mode for all the three devices.

## 2. Materials and Methods

### 2.1. Numerical Initial Method

Due to the high-cost fabrication of such complex devices, there is a strong need to accurately simulate and forecast their expected electro-optical behavior, using advanced simulations, to assure smooth functionality. The COMSOL Multi-Physics Package software (5.3, COMSOL Inc., Burlington, MA, USA) [17] is employed and integrated with Matlab-Simulink (2019, MathWorks, Natick, MA, USA) [18]. The physical equations are discretized on a mesh using the Galerkin Finite Element Method (FEM) [19] and to a reduced extent, the method of Finite Volumes (FVM). Equations can be implemented in a variety of forms, such as directly as a PDE or as a variation integral, otherwise called the weak form [19]. Boundary conditions may also be imposed directly or using variation constraint and reaction forces. Both choices have implications for the convergence and physicality of the solution. The mesh is assembled from triangular or quadrilateral elements in two-dimensions, and hexahedral or prismatic elements in three dimensions, using a variety of algorithms, pending the needs. The solution is achieved using direct or iterative linear solvers and non-linear solvers. The former is based on conjugate gradients, while the latter is generally based on the Newton–Raphson iterations.

**Figure 5:**
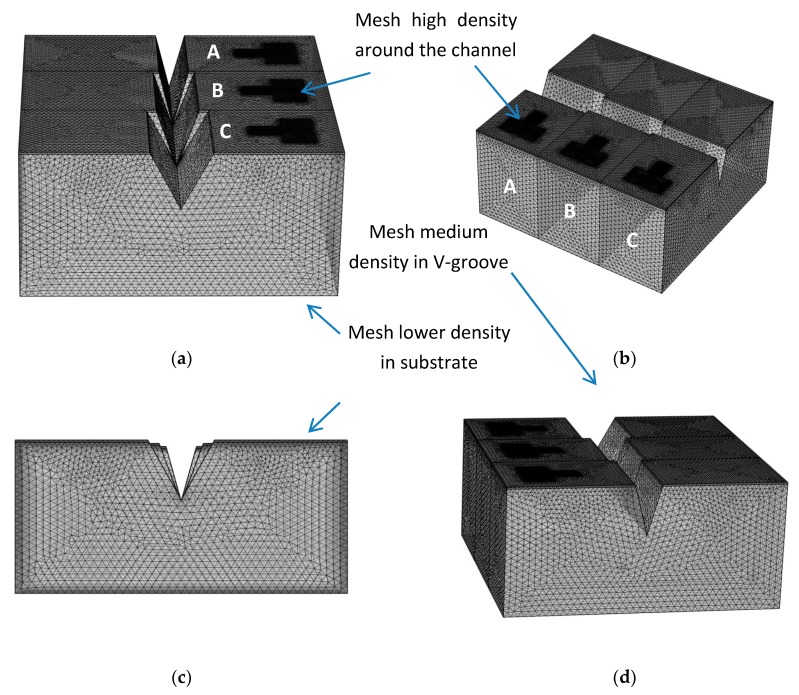
COMSOL mesh representations of the triple-SOIP^2^AM device. (**a**) Front view; (**b**) Top view; (**c**) Cross view; (**d**) Side view.

In this specific case, the required simulation models are the COMSOL “wave optics” module, and the “semiconductors” module, which are coupled during the simulations. When designing a device using the Finite Elements Method [20], and in order to perform accurate simulations, it becomes crucial to use relevant mesh elements. As presented in Figure 5, the mesh density becomes higher in the Source-Drain area, medium in the V-groove areas, and low in the substrate parts. Again, the parameters used in these simulations are summarized in Table 1.

### 2.2. Analytical Complementary Method

Until the future fabrication of more advanced generations, it is important to forecast the behavior of the new devices. Such work may require complementary analyses, such as mathematical models and numerical simulations, using COMSOL. The models may be oriented to two main directions; the time analysis and the ultra-fast polarization switch enabled with the incident beam illumination. As presented above in the introduction, the search for ultra-fast switching capability was voiced in the last decades. Enabling ultra-fast switching inside circuitry is more than desirable when compared to the continuously growing need of high-computing rate. As a consequence, a complementary analytical model is presented after the numerical results.

## 3. Simulations Results, Configurations, and Applications

The use of polarizing transistors in the combined domain of micro-electronics and electro-optics presents a lot of advantages, in particular, several major applications, as described in the following case studies. The combined new technology of light illumination with electronic data transfer will enable smart configurations of circuitry. Several case studies were analyzed.

### 3.1. Turning off the SOIP2AM by a Slight Variation of Polarization

The device default mode of work is normally ON. Assuming a single illuminated device under a fixed polarization direction, enabling to keep it ON under such a condition—an ultra-fast controlled change of the direction can enable the closure of the channel and the switch of the SOIP^2^AM single device to its OFF mode functionality.

### 3.2. Switching Mode as a Function of the V-Groove Aperture

Such a switch between the two logic modes (ON and OFF) can be dependent on the V-groove aperture (bigger or smaller than the wavelength). However, in the current study, the aperture values were chosen to fit a standard laser beam illumination diameter of few microns. Assuming a single device under constant beam illumination, the device will remain ON or will switch to OFF mode, as a function of the polarization illumination, until the incident beam changes and enables mode photo-induced current, as a function of the V-groove aperture. In these conditions, it is interesting to identify the critical dimensions in which the SOIP^2^AM device will perform the switch from the ON to the OFF modes.

**Figure 6:**
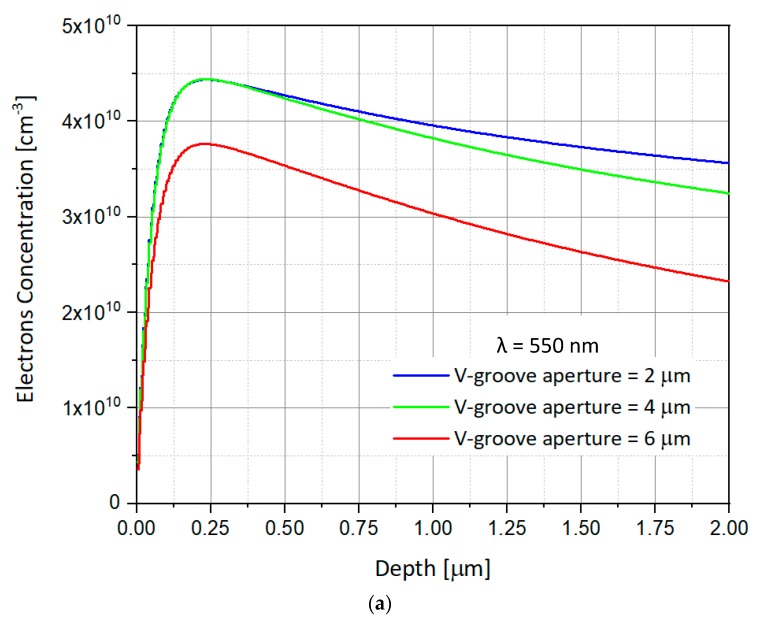

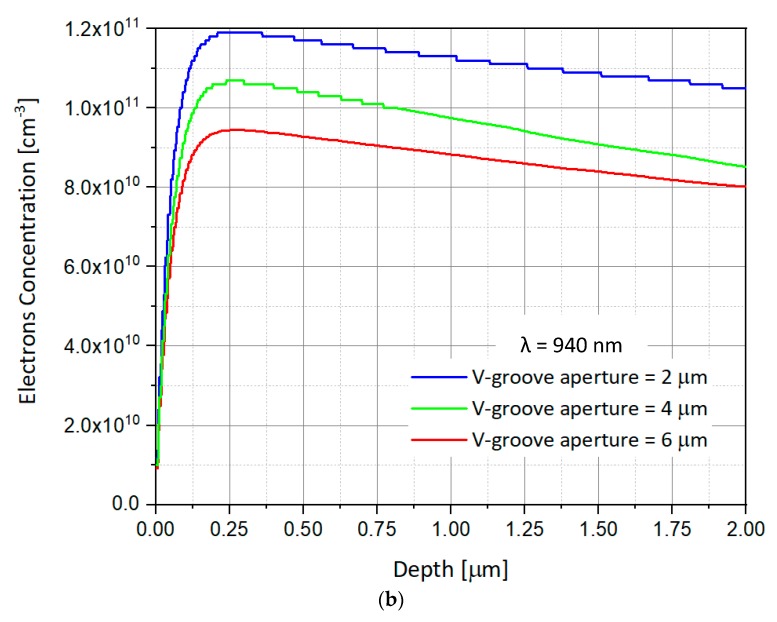
Numerical simulations showing the electrons concentration as a function of the depth in the p-substrate through the z-axis for three different V-groove apertures (2 μm, 4 μm, and 6 μm) and following the set-up: V_GS_ = −1 V, V_DS_ = 1 V, P_in_ = 10 mW. (**a**) λ = 550 nm; (**b**) λ = 940 nm.

As shown in Figure 6a, the study of the SOIP^2^AM response is presented here from three different angles α_1_, α_2_, and α_3_, and in two different wavelengths, i.e., λ_1_ = 550 nm and λ_2_ = 940 nm. If λ_1_ is usually used as a default, λ_2_ has presented two advantages. First, it allows higher efficiency in the electron-hole pairs’ generation. It also allows a more selective response of SOIP^2^AM, depending on the polarization of the illumination.

The electrons’ concentration in the silicon substrate is presented for illumination of 550 nm in Figure 6a and of 940 nm in Figure 6b, for the three different apertures of the SOIP^2^AM’s V-groove. The applied illumination power is P = 10 mW. The concentration of the electrons found in the substrate under the channel is observed as a function of the depth in the substrate. The charges diffuse throughout the substrate, and we measure the concentration very far from the V-groove. One can observe that there is a clear difference in concentrations between the different SOIP^2^AMs. The wider the opening of the V-groove, the lower the concentration is.

In the case of 550 nm, and for V-groove apertures of 2 µm and 4 µm, it reaches a maximum concentration of equivalent of 4.45 × 10^10^ cm^−3^. Also, the number of generated electrons is greater for 2 µm as evidenced by the slighter decrease in concentration according to the depth. This result makes it possible to conclude that the operation of the two SOIP^2^AMs (of respective openings 2 µm and 4 µm) should be identical; the same illumination will close the channel or not. While in the third SOIP^2^AM the reached concentrations are lower, of the order of 3.75 × 10^10^ cm^−3^ at maximum, all conditions equal. One could thus imagine a mode of operation of the Triple- SOIP^2^AM for which, at a given power, during a simultaneous illumination of all the V-grooves, the SOIP^2^AM having 2 µm and 4 µm apertures switch OFF, while the 6 µm would stay ON. By increasing the power, one could make an OFF switch of the three SOIP^2^AM. Thus, one could play ON the power of illumination to control a Triple- SOIP^2^AM without changing the position of the laser spot.

The second study presented here shares the same control parameter values as the first with the difference that this time, a wavelength illumination of 940 nm is used. Apart from the fact that the electron concentrations created by the illumination are almost tripled, one can also observe a degeneration of the maximum concentration for SOIP^2^AM with the 2 µm and 4 µm apertures, compared to the 550 nm illumination. The concentration reaches 9.4 × 10^10^ cm^−3^ for an opening of 6 µm, 1.06 × 10^11^ cm^−3^ for 4 µm, and reaches up to 1.19 × 10^11^ cm^−3^ for 2 µm. Of course, this is not a significant change. However, it lends support to its functionality. In the future, a larger study will require different apertures, when, for example, part of them share dimensions (aperture and depth) above the wavelength and the others below the wavelength.

This observation allows a methodical use of Triple-SOIP^2^AM. By illuminating the three SOIP^2^AMs, for a weak illumination power, one can make a switch OFF of only one SOIP^2^AM with a V-groove opening of 2 μm. Then gradually increasing the power, one could also turn OFF the opening of the 4 µm and 6 µm SOIP^2^AMs. In conclusion, by turning on the power, one can turn off one, two, or three devices.

### 3.3. Switching Mode as a Function of the Polarization and the Wavelength

It is now proposed to study the response of the device according to the polarization of the incident wave, and thus for the three apertures, a _V-groove_, of the SOIP^2^AM. It has been previously demonstrated [14], that the device is sensitive to the polarization conditions of the incident wave. Therefore, SOIPAM became SOIP^2^AM. This sensitivity to the polarization can allow the control of the channel’s closure by simple modulation of the incident beam’s direction. Thus, the channel of one SOIP^2^AM can remain open for specific polarization and close for another. It is also proposed to consider the previous subsection and to study this sensitivity to polarization as a function of the opening of the V-groove.

**Figure 7:**
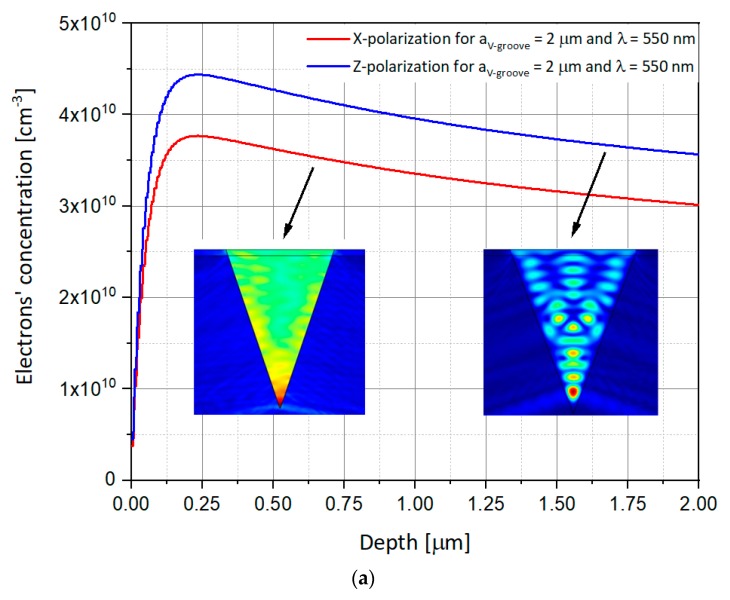

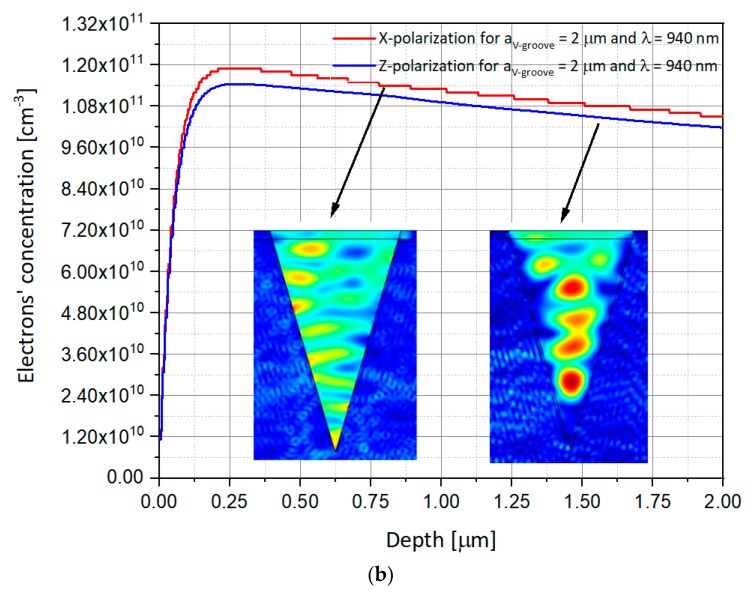
Numerical simulations showing the electron concentration as a function of the depth in the p-substrate in both the X and Z polarizations for the V-groove aperture of 2 μm. While the curves present the depth of 1D concentrations, the inserts present the 2D electric field variations inside the V-groove. The following set-up was used: V_GS_ = −1 V, V_DS_ = 1 V, P_in_ = 10 mW. (**a**) λ = 550 nm; (**b**) λ = 940 nm.

**Figure 8:**
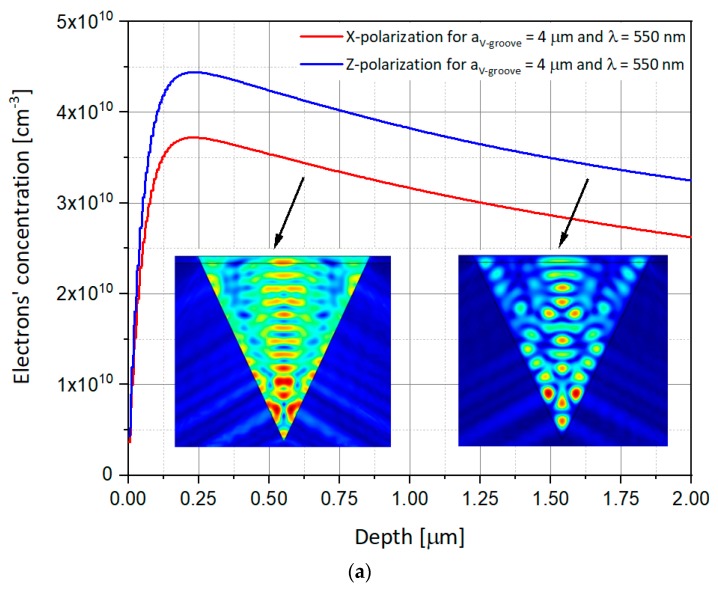

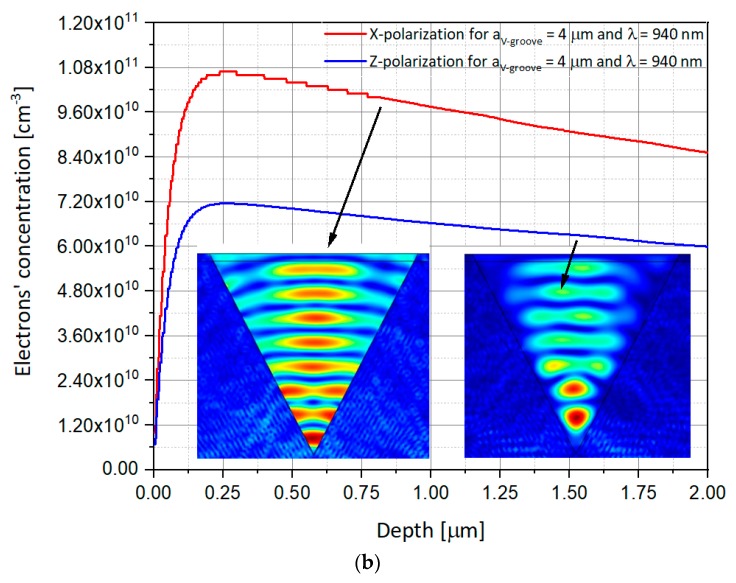
Numerical simulations showing the electron concentration as a function of the depth in the p-substrate in both the X and Z polarizations for the V-groove aperture of 4 μm. While the curves present the depth of the 1D concentrations, the inserts present the 2D electric field variations inside the V-groove. The following set-up was used: V_GS_ = −1 V, V_DS_ = 1 V, P_in_ = 10 mW. (**a**) λ = 550 nm; (**b**) λ = 940 nm.

**Figure 9:**
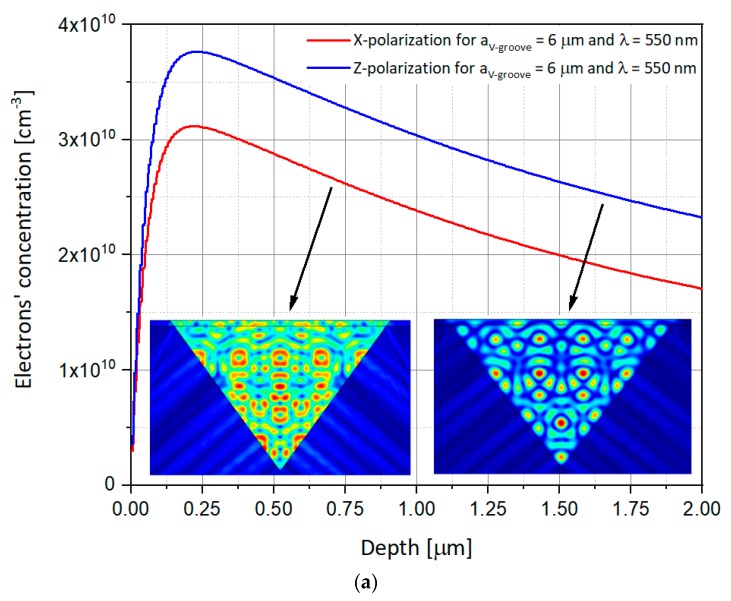

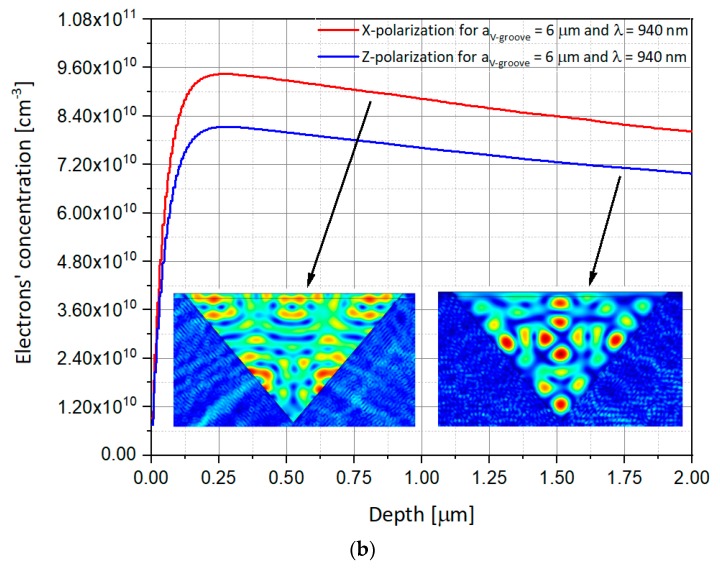
Numerical simulations showing the electron concentration as a function of the depth in the p-substrate in both the X and Z polarizations for the V-groove aperture of 6 μm. While the curves present the depth of the 1D concentrations, the inserts present the 2D electric field variations inside the V-groove. The following set-up was used: V_GS_ = −1 V, V_DS_ = 1 V, P_in_ = 10 mW. (**a**) λ = 550 nm; (**b**) λ = 940 nm.

As presented above, previous studies used the Z-polarized illumination, i.e., parallel to the V-groove vertical direction. In order to complete the analysis, it is proposed to compare the obtained results with new results, this time for polarization perpendicular to the V-groove, i.e., according to X-axis. The simulations thus use illuminations whose electric field is polarized in X or Z direction. One simulates the response of SOIP^2^AM to these illuminations for apertures of V-groove of 2 µm, 4 µm, and 6 µm, and for two different wavelengths of 550 nm and 940 nm, respectively, in order to compare the results obtained here with those of previous studies. The power of the illumination is kept constant at 10 mW, and the values of the drain and gate voltages, V_DS_ and V_GS_, are, respectively, 1 V and −1 V. The rest of the parameters are identical to those presented in Table 1. Figure 7, Figure 8 and Figure 9 show the electron concentrations in the substrate as a function of the polarization for V-groove apertures of respectively 2 μm, 4 μm, and 6 µm.

The ratio between the polarizations is very small. The ultimate expectation would be to see a ratio of about one order of magnitude at least; otherwise, it is not a significant polarization sensitivity (a polarizer gives two orders of magnitude difference and more). This is because the aperture of the channel should be in one axis sub-wavelength, and in the other, more than the wavelength. The depth should also be several wavelengths in order for the evanescent waves not to generate electrons.

Some important observed phenomena from Figure 7, Figure 8 and Figure 9 may require special attention:

- Once again, the electron concentrations in the Si substrate are much higher, at the same opening size, for applied λ_2_ = 940 nm when compared to λ_1_ = 550 nm, whatever would be the polarization. This can be easily explained by the spectral responsivity of silicon [21].
- Moreover, if for a wavelength illumination of 550 nm, the electrons’ concentration curve at X-polarization remains lower than the Z-polarization’s one, the opposite phenomenon is observed for an illumination of wavelength 940 nm, for all the V-groove apertures. This may be explained by the interference figure close to the silicon groove edge which is destructive for Z polarization at 940 nm while constructive at 550 nm.
- Regarding such reversed concentration, one can use this advantage to reverse the SOIPAM operation mode of work, by changing the wavelength, enabling the device to stay closed for polarization in the X direction, and opened in the Z one.

Such interesting observations suggest new possibilities of integration of X-SOIP^2^AM. Indeed, as it was mentioned, by changing the polarization, one can choose to turn off or not a SOIP^2^AM. Indeed, for illumination of 550 nm, one will be able to design the SOIPAM so that the SOIPAM of an aperture of 4 µm (and can be even 6 µm), does not go out with polarization in X but go out with polarization in Z. Regarding the SOIP^2^AM with an aperture of 2 µm, the curves of different polarization being superimposed, it can switch OFF no matter the polarization condition. As a preliminary conclusion, it appears that the ON/OFF mode can be selectively controlled by the modulation of the polarization and that adjacent devices can act on a binary basis as part of a circuitry. These results enable exploring large and versatile usage of X-SOIP^2^AM devices.

### 3.4. Carriers Distribution in Slice Planes

**Figure 10:**
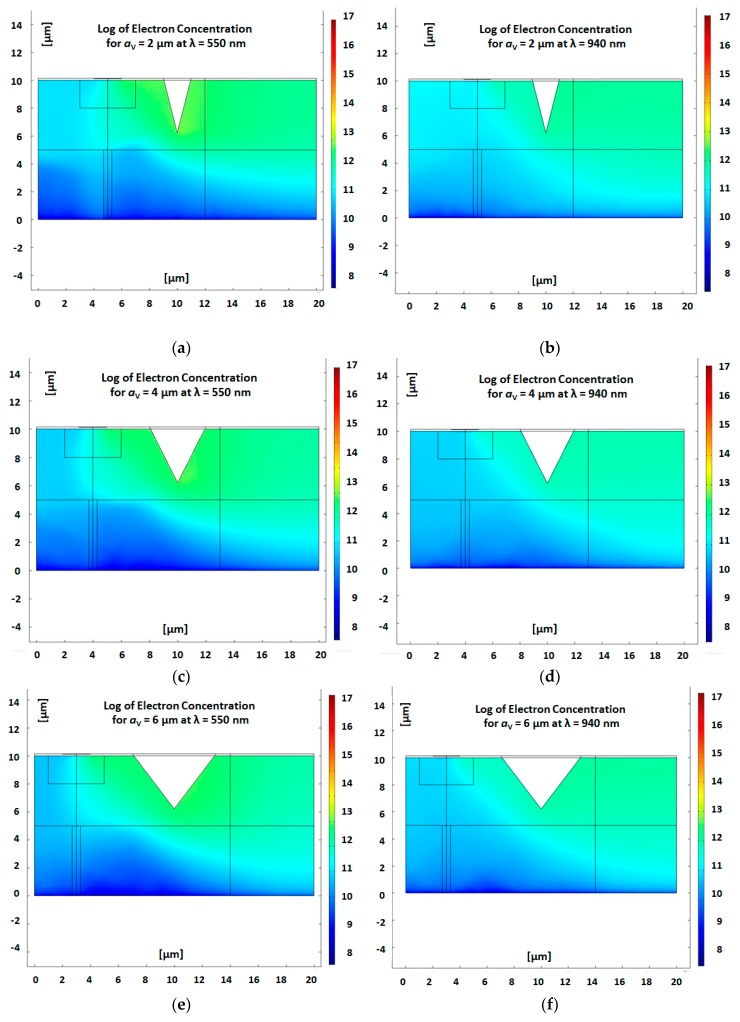
COMSOL numerical simulations, showing the 2D electrons concentration in the p-substrate for several V-groove apertures and wavelengths. (**a**) a _V-groove_ = 2 µm (28.48°), λ_1_ = 550 nm; (**b**) a _V-groove_ = 2 µm (28.48°), λ_2_ = 940 nm; (**c**) a _V-groove_ = 4 µm (53.83°), λ_1_ = 550 nm; (**d**) a _V-groove_ = 4 µm (53.83°), λ_2_ = 940 nm; (**e**) a _V-groove_ = 6 µm (74.57°), λ_1_ = 550 nm; (**f**) a _V-groove_ = 6 µm (74.57°), λ_2_ = 940 nm. In all cases, V_GS_ = −1 V, V_DS_ = 1 V, P_in_ = 10 mW.

In order to get the profile of the carrier’s distribution (both electrons and holes), inside and outside the channel, it was necessary to fix a specific cross-section slice across the device. In such a way, there is a possibility to obtain the distribution curves as a function of the geometry. In Figure 2, which is the 2D extension of the 1D profile shown below, several cross-sections of investigations were presented, and their corresponding obtained profiles are now presented in Figure 10.

### 3.5. Adjacent vs. Perpendicular Devices

The last configuration of SOIP^2^AM devices was checked; this time, the devices are perpendicular and not parallel. The polarization analysis focuses on a larger incident circular beam capable of illuminating all the devices at the same time. As a consequence of their geometry, dimensions, and orientation, part of the devices moves to the OFF mode, while another part remains normally in the ON default working mode.

## 4. Discussion and Complementary Analytical Model

In order to enable an enhanced and solid analysis, several points need to be emphasized in the perspective of previous studies and of the working hypotheses. First, it was important to follow up on the device evolution and planned roadmap, then to discuss the red hot spots results and how they can be interpreted, and finally, to present a complementary analytical model, which fits and completes the numerical simulations.

### 4.1. MOSFET, SOIPAM, SOIP2AM, and N-SOIP2AM Evolution and Roadmap

In an overview of the MOSFET transistors’ geometry evolution, one can look at the different generations; starting from the well-known standard MOSFET transistor (Source, Gate, and Drain all located in the same plane), moving forward to the SOIPAM device (Gate located in the bulk, and serving as an intensity modulator), continuing through the SOIP2AM device (serving not only as an intensity modulator, but also as a polarization one), and finishing with the N-SOIP2AM (enabling complex circuitry of ON/OFF devices). The integration of such devices may enable polarizing transistors, acting selectively as a function of the X, Y polarization directions. Modifying the CMOS architecture with minor changes (additional masks and lithography), such modulators can easily be combined in a smooth way inside a standard circuitry.

### 4.2. Interferences Inside the Structure

A phenomenon that needs to be explained has been mentioned above—the decrease in the concentration of electrons in the substrate when the opening of the V-groove increases. In order to understand this phenomenon, one can observe the figures of the electric field variation in the V-groove. To explain this, it is proposed to rely on the observation of the presence of interferences within the V-groove as shown in the following figures. One can see that when the aperture of the V-groove decreases, the number of maximal intensities of the electric field (red hot spots) decreases (three maximums for 6 µm, then two maximums for 4 µm, and finally one unique maximum for 2 µm). However, the intensity of these maxima increases and it is observed that the maxima of the 6 µm are not glued to the substrate but are in the air. These are potential centers of “not used” generated electron-hole pairs production, therefore, for the 6 µm, unlike what happens in 4 µm case or 2 µm case, where the maxima are well glued to the substrate, and therefore certainly produce a lot of generated pairs of electrons holes. The interference phenomenon has been presented in the inserts of Figure 7, Figure 8 and Figure 9.

### 4.3. Simulation vs. Modeling—The Complementary Analytical Model

Until the fabrication of the next generations, it was important to forecast the behavior of the new devices. Such work may require complementary analyses, such as mathematical models and numerical simulations, using COMSOL. The models may be oriented to two main directions: the time analysis and the ultra-fast polarization switch enables with the incident beam illumination. As presented above in the introduction, the need for ultra-fast switching capability has been searched in the last decades. Enabling ultra-fast switching inside circuitry is more than desirable when compared to the continuously growing need of high-computing rates.

Starting with Maxwell equations:

(2) $$\bar{\nabla }\;\times \bar{E}\left(\bar{r}\right)\;=\;i\omega \mu \left(\bar{r}\right)\bar{H}\left(\bar{r}\right),$$

(3) $$\bar{\nabla }\times \bar{H}\left(\bar{r}\right)\;=\;G\left(\bar{r}\right)\bar{E}\left(\bar{r}\right)\;-\;i\omega \epsilon \left(\bar{r}\right)\;\bar{E}\left(\;\bar{r}\right),$$

where E and H are the electrical and the magnetic fields. ϵ and μ are the dielectric constant; the magnetic permeability, and G is the conductivity. ω is the radial frequency of light.

Following the structure of our devices having aperture being narrow in x and long in y (sub-wavelength in x and with the length of several wavelengths in y), we will assume that the symmetry in y yields ∂/∂y = 0. Then, for E_y_ one obtains [22,23]:

(4) $$-\frac{\partial }{\partial z}E_{y}\left(x,z\right)\;={\;i\omega \mu }_{0}H_{x}\left(x,z\right),$$

(5) $$-\frac{\partial }{\partial x}E_{y}\left(x,z\right)\;={\;i\omega \mu }_{0}H_{z}\left(x,z\right),$$

It results that:

(6) $$\frac{\partial }{\partial z}H_{x}\left(x,z\right)\;-\;\frac{\partial }{\partial x}H_{z}\left(x,z\right)\;=\;G\left(x,z\right)E_{y}\left(x,z\right)\;-\;i\omega \epsilon \left(x,z\right)E_{y}\left(x,z\right),$$

where assuming that $\mu \;={\;\mu }_{0}.$

And for E_x_, it results:

(7) $$-\frac{\partial }{\partial z}E_{x}\left(x,z\right)\;={\;i\omega \mu }_{0}H_{y}\left(x,z\right),$$

(8) $$-\frac{\partial }{\partial z}H_{y}\left(x,z\right)\;=\;G\left(x,z\right)E_{x}\left(x,z\right)\;-\;i\omega \epsilon \left(x,z\right)E_{x}\left(x,z\right),$$

One can see that the differential equations for E_y_ and E_x_ are different, and thus, light will be polarized. To solve these differential equations, one will perform ∂/∂z and substitute one equation into the other. This yields to:

(9) $$\frac{\partial^{2}}{\partial z^{2}}E_{y}\left(x,z\right)\;+\;\frac{\partial^{2}}{\partial x^{2}}E_{y}\left(x,z\right)\;+\;\left(\omega^{2}\mu_{0}\epsilon \left(x,z\right)\;+{\;i\omega \mu }_{0}G\left(x,z\right)\right)E_{y}\left(x,z\right)\;=\;0,$$

And

(10) $$\frac{\partial^{2}}{\partial z^{2}}E_{x}\left(x,z\right)\;+\;\left(\omega^{2}\mu_{0}\epsilon \left(x,z\right)\;+{\;i\omega \mu }_{0}G\left(x,z\right)\right)E_{x}\left(x,z\right)\;=\;0,$$

Since:

(11) $$\epsilon_{r}\left(x,z\right)\;={\;n}^{2}\left(x,z\right)\;=\;\frac{\epsilon \left(x,z\right)}{\epsilon_{0}}\;+\;\frac{\mathrm{iG}\left(x,z\right)}{\omega \epsilon_{0}},$$

And

(12) $$k\;=\;\frac{2\pi }{\lambda }\;=\;\omega \sqrt{\epsilon_{0}\mu_{0}},$$

With λ being the optical wavelength, it results:

(13) $$\frac{\partial^{2}}{\partial z^{2}}E_{y}\left(x,z\right)\;+\;\frac{\partial^{2}}{\partial x^{2}}E_{y}\left(x,z\right)\;+{\;k}^{2}\epsilon_{r}\left(x,z\right)E_{y}\left(x,z\right)\;=\;0,$$

And

(14) $$\frac{\partial^{2}}{\partial z^{2}}E_{x}\left(x,z\right)\;+{\;k}^{2}\epsilon_{r}\left(x,z\right)E_{x}\left(x,z\right)\;=\;0,$$

Assuming that the diffraction after the aperture is done in free space, and thus, $\epsilon_{r}\;=\;1$, the solution of the differential equation for E_x_ gives:

(15) $$E_{x}\left(x,z\right)\;=\;\exp\left(-\mathrm{ikz}\right),$$

This means that the radiation is back-reflected, and it is not propagated into the aperture. To solve the differential equation for E_y_, a Fourier transform will be performed over the x-coordinate, and it will result:

(16) $$\frac{\partial^{2}}{\partial z^{2}}{\;\tilde{E}}_{y}\left(\mu ,z\right)\;-\;{\left(2\pi \mu \right)}^{2}{\;\tilde{E}}_{y}\left(\mu ,z\right)\;+{\;k}^{2}{\;\tilde{E}}_{y}\left(\mu ,z\right)\;=\;0,$$

This leads to a solution of:

(17) $${\;\tilde{E}}_{y}\left(\mu ,z\right)\;=\;{\;\tilde{E}}_{y}\left(\mu ,0\right)\exp\left(\mathrm{ikz}\sqrt{1\;-{\;\lambda }^{2}\mu^{2}}\right),$$

Which means that spatial frequencies which are lower than µ < 1/λ propagate as harmonic waves while the high spatial frequencies above µ > 1/λ are becoming evanescent waves decaying with z.

Thus, if the device contains a series of V-grooves each oriented in a different location, then the largest electrical current will occur when the direction of the aperture of each V-groove matches the axes of the linear polarization of the illuminating beam at the spatial location of that V-groove (to have the x and y of the polarization indeed coincide with the short and long axes of the V-groove). This means that the proposed set of devices can be used for the application of encryption/decryption device since the strongest electrical responsivity will be obtained only for a very specific spatial polarization distribution of the illumination beam in which the direction of the aperture of each V-groove will coincide with the axes of the linear polarization of the illuminating beam at the spatial location of that V-groove (the x and y of the polarization coincide with the short and long axes of the V-groove) [23].

## 5. Conclusions

Several circuitry configurations of polarizing transistors were studied in order to check the ultra-fast switching mode as a function of important parameters such as λ—the wavelength of the incident illumination beam, α _V-groove_—the angle of the V-groove aperture, and *E_X,Y_* the polarized field direction. It appears that combining two, three, and more coupled devices enables building logic gates, modulating not only the intensity of the photo-induced current but also the polarization. Such novelty can enable in the future a new generation of integrated electro-optics coupled devices inside the existing silicon technologies of the microelectronics industry.

Moreover, the proposed set of devices can be used for the application of encryption/decryption device since the strongest electrical responsivity will be obtained only for a very specific spatial polarization distribution of the illumination beam in which the direction of the aperture of each V-groove will coincide with the axes of the linear polarization of the illuminating beam at the spatial location of that V-groove (the x and y of the polarization coincide with the short and long axes of the V-groove).

## Figures and Tables

**Table 1:** SOIPAM device parameters.

Parameter	Definition	Value
**Device parameters**		
W _Silicon_	Substrate width	20 µm
L _Silicon_	Substrate length	7 µm
H _Silicon_	Substrate height	10 µm
L _V-groove_	V-groove length	2.78 µm
H _V-groove_	V-groove height	3.94 µm
t _box_	BOX thickness	150 nm
t _n_ = t _channel_	Channel thickness	30 nm
L _channel_	Channel length (Source-Drain)	300 nm
W _channel_	Channel width	2 µm
N_D_	n-doped channel donors (P) concentration	~1.10^17^ cm^−3^
N_A_	p-doped substrate acceptors (B) concentration	~1.10^15^ cm^−3^
**The setup parameters:**		
V_G_	Gate voltage	−1 V
V_S_	Source voltage	0 V
V_D_	Drain voltage	1 V
P _in_	Laser power	10 mW
λ	Wavelength	550 nm, 940 nm
a _V-groove_	V-groove aperture	a_1_ = 2 µm, a_2_ = 4 µm, a_3_ = 6 µm
α _V-groove_	V-groove angle	α_1_ = 28.48°, α_2_ = 53.83°, α_3_ = 74.57°
**Measured parameters:**		
$n_{s}^{max}$	Maximum electron concentration in substrate	3.75 × 10^10^ to 1.19 × 10^11^ cm^−3^
